# Indirect Selection against Antibiotic Resistance via Specialized Plasmid-Dependent Bacteriophages

**DOI:** 10.3390/microorganisms9020280

**Published:** 2021-01-29

**Authors:** Reetta Penttinen, Cindy Given, Matti Jalasvuori

**Affiliations:** 1 Department of Biological and Environmental Science and Nanoscience Center, University of Jyväskylä, Survontie 9C, P.O.Box 35, FI-40014 Jyväskylä, Finland; reetta.k.penttinen@jyu.fi (R.P.); cindy.j.given@jyu.fi (C.G.); 2 Department of Biology, University of Turku, FI-20014 Turku, Finland

**Keywords:** antibiotic resistance, conjugative plasmids, plasmid-dependent, male-specific, pilus-binding, bacteriophages

## Abstract

Antibiotic resistance genes of important Gram-negative bacterial pathogens are residing in mobile genetic elements such as conjugative plasmids. These elements rapidly disperse between cells when antibiotics are present and hence our continuous use of antimicrobials selects for elements that often harbor multiple resistance genes. Plasmid-dependent (or male-specific or, in some cases, pilus-dependent) bacteriophages are bacterial viruses that infect specifically bacteria that carry certain plasmids. The introduction of these specialized phages into a plasmid-abundant bacterial community has many beneficial effects from an anthropocentric viewpoint: the majority of the plasmids are lost while the remaining plasmids acquire mutations that make them untransferable between pathogens. Recently, bacteriophage-based therapies have become a more acceptable choice to treat multi-resistant bacterial infections. Accordingly, there is a possibility to utilize these specialized phages, which are not dependent on any particular pathogenic species or strain but rather on the resistance-providing elements, in order to improve or enlengthen the lifespan of conventional antibiotic approaches. Here, we take a snapshot of the current knowledge of plasmid-dependent bacteriophages.

## 1. Introduction

Antibiotic resistance has steadily increased across the world mostly due to the use of antibiotics. The lack of effective treatments against bacterial infections compromises the use of various medical procedures such as organ replacements and chemotherapies where bacterial infections are kept at bay with antibiotics [1]. At the heart of the resistance problem is natural selection: we kill those bacteria that are unable to cope with the presence of antibiotics, leaving behind a collection of survivors [2]. As we apply more and different antibiotics at the remainder, the selective process is repeated. Eventually we are left with only resistant bacteria. However, intuitive lines of thought often go wrong when we attempt to construe how the resistance factualizes in reality [3]. 

**Figure 1:**
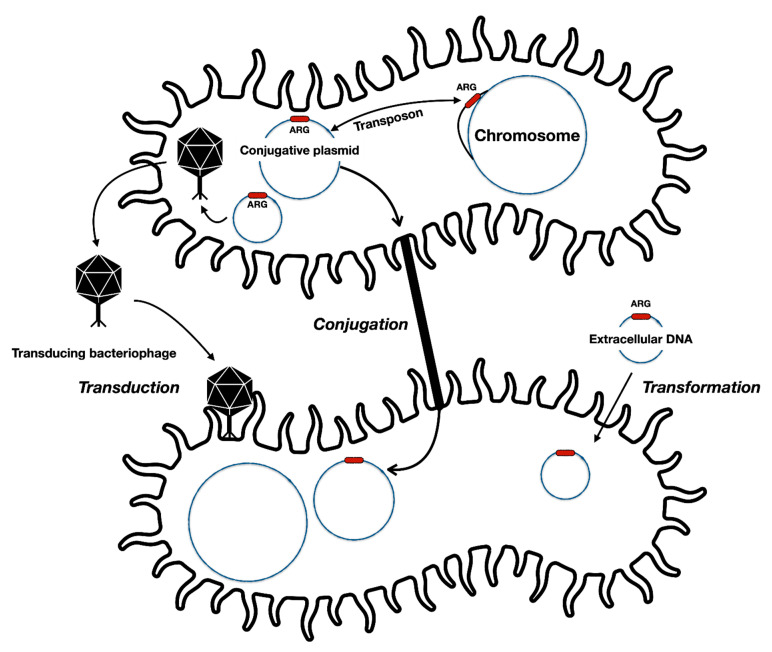
General forms of antibiotic resistance gene (ARG) transfer between bacterial cells and different genetic elements. Resistance providing genes may be transferred along with transposing elements from chromosomes to plasmids and vice versa. Some bacteriophages may accidentally package resistance genes into their capsids and later infect other susceptible cells. Some bacteria can also uptake DNA from the environment and incorporate them into the chromosome.

It would seem that resistance develops gradually, and bacteria come up with their own *de novo* solution (such as mutations that modify the target site) to constrain the effects of the antibiotic, one by one. Bacteria acquire mutations that modify the targets of antibiotics and hence nullify their effectiveness. This is what we observe when isolated bacterial cultures are exposed to antimicrobials [2]. In practice and in environments where it has a notable effect (such as hospitals), however, bacteria most often receive a complete and efficient antibiotic resistance directly from their contemporaries instead of deciphering the evolutionary solution via mutations [4,5]. The resistance mechanism itself is often different from gradually developed ones, such as enzymes that hydrolyze antibiotic molecules [6]. Resistance may be acquired via, e.g., transformation (i.e., uptake of genetic material from the environment) or transduction (i.e., within genome-integrating bacteriophage that contain resistance genes) (Figure 1). However, at least for problematic Gram-negative bacteria, the transfer of resistant phenotypes is frequently mediated by mobile genetic elements such as conjugative plasmids that have no loyalty to any particular bacterial species or strain [7]. From the gene’s point of view, it is irrelevant who the host is as long as the gene itself prevails [8]. When antibiotics are present, the eye of selection fixes on those who are not masked by a resistance. Yet, it is alerting that a single conjugative plasmid (such as PA1705-NDM) can carry resistance genes against most if not all antibiotics [5]. Therefore, in an environment where only a single antibiotic is present, a plasmid that provides resistance to a variety of antibiotics can become dominant. In other words, nearly complete evolution of resistance to all drugs can occur in an instant even if such an evolutionary jump would appear completely impossible in a test tube [5].

Antibiotic resistance genes are opportunistic in a sense that they provide immense benefit under specific conditions (presence of antibiotics) but are otherwise just a slight burden in terms of reduced replication rate for the host [8,9,10]. However, studies have shown that the burden is not enough to reverse our artificially introduced selection for resistance even if we actually could retain from using antibiotics [11]. Is it possible to make the resistance more costly to the bacterium and hence accelerate its lost? This may be difficult to achieve unless we can somehow target the particular gene or its product within the host cell. Instead, it may be more efficient to target the mobile genetic element and put their carriers and the ability to disperse horizontally at disadvantage within bacterial communities. 

Encouraging bacteria to drop their plasmids is possible by at least three distinct mechanisms. First, bacterial cultures can be introduced with molecules that destabilize plasmid separation, conjugation or replication and hence improve the chances for plasmid to get cured from the community [12,13]. Second, plasmid DNA-sequence may be targeted with CRISPR/Cas9 system. CRISPR-system is originally a bacterial defence mechanism that has later on been adapted for various genetic engineering purposes. By introducing Cas9 together with specific guiding RNA into bacterial cell, the endonuclease nicks the double stranded plasmid DNA [14,15,16]. Linearized plasmid cannot be replicated normally, leading to either the loss of plasmid or, in case of plasmid encoded addictive toxin-antitoxin systems, even bacterial death. The third strategy to cure plasmids utilizes bacteriophages (phages), viruses that infect bacteria [17]. In this discussion we focus on the last one and go through the potential ways by which phages could be harnessed against antibiotic resistance.

## 2. Plasmid-Dependent Bacteriophages 

There are bacteriophages that target plasmid-encoded structures (such as a pilus) on bacterial cell surface. This, in turn, makes the viruses dependent on particular plasmids in the bacterial cell. In practice, the virus has evolved to recognize features that are transcribed from genes that reside in the plasmid instead of bacterial chromosome. Naturally, the phage still infects bacterial cells, but only when it carries a plasmid with a gene for phage-exploited receptor. Interestingly, these phages are generally completely different from the abundant head-tail viruses of Caudovirales and as such they seem to have adapted to this specialized plasmid-dependent lifestyle for a long time, some possibly even for the entirety of existence of bacteria on Earth [10,18,19]. Previously, one of the authors and Eugene Koonin devised a classification for genetic replicators in bacterial cells based on the replicator’s dependency and cost on any particular cell and their mode of mobility [8]. One of the classes was conjugative elements (others were chromosomes, non-mobile and mobile plasmids and different types of viruses) and as such the plasmid-dependent viruses could be seen as parasites of plasmids rather than chromosomes. Yet, in reality the borders between classes are elusive and may evolve to become one of the other groups. Still, plasmids are ancient and the mobile framework for opportunistic genes can provide specialized viruses a unique niche within the bacterial realm. 

Tectivirus PRD1 is one of the best-studied plasmid-dependent (also known as male-specific) bacteriophages [20]. It has an icosahedral protein capsid of size ~60 nm with an inner membrane [21]. During an infection, PRD1 binds to the mating-pair formation apparatus (encoded by some IncP, IncW and IncN plasmids) on the bacterial cell [22]. Once bound, the phage injects its double-stranded DNA genome via a lipid tube through the cell wall and into the cytoplasm [23,24]. Therein, the phage makes copies of its DNA and produces a variety of structural proteins that eventually assemble into complete capsids [25]. At the final stages of infection, the double stranded DNA is packaged into the capsid by an ATPase and the lytic enzymes and holins together cause the lysis of the bacterial cell and the release of phage particles into the environment [26]. A single iteration of a PRD1 life-cycle can generate hundreds of new viruses, each of which is capable of infecting another cell that carries a suitable plasmid. For PRD1, the methodology for conducting in vitro genome packaging has been developed [27,28]. The genome contains a terminal protein that is both used to initiate DNA replication and genome translocation into preformed capsids. As such, it could be possible to utilize PRD1 capsids to deliver DNA-cargo (such as CRISPR-Cas9 system) into plasmid-harboring (and therefore resistant) bacteria. However, to our knowledge, this approach has not been experimentally tested.

The inovirus M13 is a well-studied representative of Ff class filamentous phages (including phages f1 and fd) that are specific to the conjugative F plasmid [29,30]. The infection process of Ff phages have been studied extensively (see, e.g., [31,32] for review). Briefly, the phage attaches to the tip of the F pilus [30], and by the retraction of the pilus, the phages are pulled closer to the bacterial cell [33]. On the cell surface, the phage interacts with a membrane-associated protein complex TolQRA [34,35]. The virion proteins are relocated onto the host inner membrane [36,37], releasing the single stranded phage DNA into the cytoplasm where the production and assembly of new capsids occurs. However, in contrast to PRD1, M13 does not lyse the cell but keeps secreting phage particles practically indefinitely (see, e.g., [31]). M13 has been an important tool in biotechnology as the phage genome can be easily engineered to carry desired (recombinant) genetic sequences that are incorporated into the virus particle (virion) [38]. This phage display technology provides a simple access to produce proteins for a variety of uses, such as antigen display for generating antibodies [39].

Leviviruses (such as the well-studied MS2) are small (26 nm in diameter) icosahedral positive-strand RNA viruses that, similarly to M13, use the F pilus for host attachment but bind along the sides of pilus (see, e.g., [40,41]). New leviviruses are released via lytic lifecycle. Levivirus Qβ has been used to demonstrate in vitro evolution of genetic material in the absence of any living entities [42]. The continuous selection eventually reduced the phage genome only into the replication starting site (so-called Spiegelman’s monster after Sol Spiegelman) [43]. This demonstration has served for a narrative for various discussions revolving around the origin of life and genetic material.

Altogether, the known plasmid-dependent (or male-specific) bacteriophages target a wide range of plasmids with different incompatibility (Inc) types (exemplified in Table 1), including the most prevalent types (such as IncF, IncH, IncI) associated with antibiotic resistance genes [44]. These phages appear to be relatively common in human and domesticated animal feces as well as in sewage [45,46]. However, isolation of new plasmid-dependent phages can be challenging, if there is an abundance of phages that target chromosome-encoded receptors. Yet, exposing the enrichment source (such as sewage sample) briefly to a bacterial host lacking the plasmid, the phages with “conventional” receptors may be pulled out and hence proportionally increase the remaining plasmid-dependent phages [47]. The host range of plasmid-dependent phages generally, yet not always (see, e.g., [48]) aligns with that of the plasmid. In other words, if the plasmid is able to replicate in a particular host, then the phage is likely to be able to infect that host (if it carries the plasmid). It is also important to denote that phages specific to a particular incompatibility group are still unlikely to infect most of the plasmids in a particular incompatibility group. Rather, plasmids and phages are supposed to also coevolve in nature comparably to other phages and bacteria. In other words, those plasmid-encoded features that allow phage-infection are likely to diverge to avoid phage attachment, and correspondingly, phages evolve to recognize altered plasmid-encoded proteins. However, to our knowledge, there are no direct experimental demonstration of such coevolution occurring within in vivo communities. Yet, comparison of genomes of plasmid-dependent phages shows that variation among genes for receptor binding proteins is higher compared to other regions [49,50], suggesting that evolutionary arms race is true also for these phages.

## 3. How Does Plasmid-Dependent Phages Affect Resistant Bacteria?

Introduction of plasmid-dependent phages to an environment, where there are suitable plasmids and their host bacteria, have several effects on plasmids and their hosts. Presence of lytic, plasmid-dependent phages favors bacteria that do not carry plasmids and are therefore unrecognized by phage’s host recognition proteins. This can lead to at least partial “plasmid curing” from the community as, for example, PRD1 selection in vitro resulted in vast majority of *Escherichia coli* and *Salmonella enterica* serovar Typhimurium hosts harboring IncP and IncN conjugative plasmids to become plasmid-free [74]. Similarly, F-plasmid-specific MS2 causes *E. coli* and *Salmonella* Enteritidis to drop plasmids [75]. Furthermore, PRD1 efficiently prevents plasmid transfer and hence antibiotic resistance genes between two bacterial strains even in conditions that strongly promote plasmid exchange in the community [76]. In other words, the plasmid provides a resistance for a particular bacterium that is superior to other bacteria in the environment. Yet, plasmid-dependent phage prevents this at least for a while. Additionally, simultaneous selection against (via plasmid-dependent phage) and for (via antibiotics) conjugative plasmid forces the bacteria to retain the plasmid but it generates (favors) plasmid-mutants that are unable to conjugate. Given that antibiotic resistance plasmids may be transferred to infective agent (pathogen) only when the antibiotic treatment starts (or even afterwards [74]), plasmid-dependent phages can prevent these types of evolutionary rescue events from taking place in cases where patients have been identified to be, e.g., ESBL-carriers [77]. Similar to PRD1, MS2 prevents conjugation between bacteria [75]. Ff phages that bind to the tip of pili also impair the pilus-induced biofilm formation [78].

Overall, selection in vitro against conjugative plasmid generates plasmid-free (antibiotic susceptible) cells and, with PRD1, a small fraction of plasmid mutants that have either very low or completely abolished conjugation ability [17]. Those mutants that have reduced conjugation rate may sometimes revert back to the original phenotype, but this also restores their susceptibility to PRD1. The reversible mutations were identified to be due to tandem-repeat additions, which are known to be dynamic (i.e., they can disappear rapidly from the genetic material during replication). Therefore, it is possible that under conditions where reversibility is highly favorable for plasmid (and bacterial) survival, dynamic mutations allow plasmids temporarily protect their hosts from phage infections but restore full conjugation ability once the phage disappears from the environment. Nevertheless, phage selection does also interfere with long-term plasmid survival as demonstrated in a 50-day serial culture experiment [3]. However, in vitro studies may not directly translate into in vivo efficacy. Within an insect larva gut, the highly structured environment appears to only moderately limit the dispersal of plasmids and/or enumeration of antibiotic resistant bacteria in the presence of PRD1 [79]. This could be due to the poor access that phages have to the plasmid-harboring cells in the gut or due to immune defences that restrict phage. Yet, in chickens, the F-plasmid specific MS2 can prevent the spread of *Salmonella* Enteritidis (harboring F-plasmid) infection among birds and modify the once-resistant bacterial population towards susceptibility to antibiotics [75]. Clearly, however, more research is needed to see whether plasmid-dependent phages can become a relevant tool to supress the dispersal of opportunistic traits.

## 4. Dam the Flow: Why Disrupting Genetic Exchange Can Make a Difference?

**Figure 2:**
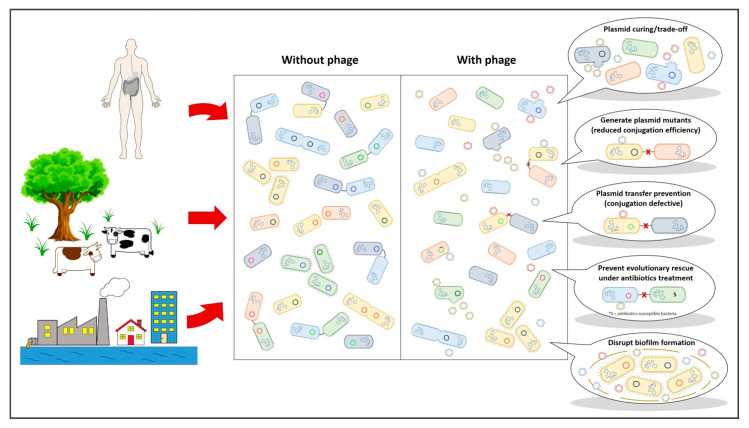
Plasmid-dependent bacteriophages can have a multitude of beneficial effects within bacterial systems. S = antibiotics-susceptible bacteria.

As of now, we have allowed bacterial communities to manifest all their tools to overcome antibiotics. Genetic exchange between distantly related cells have made it possible for initially rare but highly beneficial characteristics to spread from one bacterium to another. The selective process on a community level have steadily filtered the most useful genes into mobile genetic backbones [80,81]. These backbones, however, are still not without their own trade-offs when it comes to survival in microbial systems [11,82]. Lopatkin and colleagues demonstrated the delicate dependency on conjugation rate and plasmid survival even in the absence of antibiotic selection for plasmid-borne resistance genes [11]. Still, our continuous administration of antibiotics has evened out most of the possible fitness caveats and we have lacked means to dam the process. Plasmid-dependent phages are clearly one such means by which the downsides of plasmid-carrying can be pronounced. Therefore, even if phage-mediated selection is not immediately eradicating plasmids, they can still interfere the “natural” order of things in the microbial realm where evolution towards resistance becomes reality (Figure 2). Given the potential importance of conjugation rate on plasmid-prevalence, even a small decrease in the frequencies by which plasmids disperse may generate beneficial effects. As such, they may help prolong the efficacy of current antibiotics and stall the development of resistance against new antimicrobials.

In nature, bacteria face various threats such as competition, predation or different types of antimicrobials against which resistance mechanisms can be either altruistic (i.e., bacteria can protect their contemporaries) or selfish (i.e., the bacterium protects only itself). In the presence of an antibiotic for which altruism (or more precisely ‘leaky’) resistance is possible, plasmid-dependent phage forces only the necessary minority to harbor resistance plasmids [3]. In the absence of the phage, the whole community stably maintained the resistance. This suggests that even in environmental settings where antibiotics are selecting for resistance, we may be able to limit the prevalence of resistance elements, which, in turn, may result in reduced transmission of plasmid from the community to pathogens. However, when the system contains sublethal concentrations of antibiotics where resistance only protects the host, the resistance-providing plasmids prevail. The resistance, even if the antibiotics only hinder bacterial growth, is of such fitness advantage that selection via plasmid-dependent phages do not favor plasmid-curing. Yet, much of the plasmid-harboring cells do become conjugation deficient. Overall, this suggests that environments where there are antibiotics present against which selfish-resistance is predominant, plasmid-dependent phages are likely to provide a less effective curing effect [3,83].

Interestingly, efficient conjugation appears to be necessary for plasmids to prevail when bacteria serve as prey for protozoa [84]. Predation increases the metabolic rate in bacteria, which in turn may require the plasmids to be able to disperse between cells. As such, trophic interactions that are abundant in environmental reservoirs can help accelerate plasmid-loss when plasmid-dependent phages are used to select for conjugation deficient mutants. Moreover, reducing bacterial tendency to form resilient biofilms with pilus-binding phages [78], the development of resisting reservoirs of pathogens and/or resistance plasmids may be tempered to some extent.

## 5. Concluding Remarks

To conclude, plasmid-dependent phages are currently almost completely unexplored option to fight antibiotic resistance. In the past, plasmid-dependent phages were isolated in great numbers by several research groups, but recently much of the phage-therapy practices has focused solely on species- or strain-specific tailed phages. Given the current realization of the problems that antibiotic resistance may induce in the society, the importance of plasmids as a player in the problem and the re-emergence (or makeover) of phages as a possible solution (in some cases) [85], plasmid-dependent phages deserve yet another look. Much of the previous work has been done in terms of basic virology and they have revealed significant insights to the evolution of viruses on our planet [18,19,21], but these phages arguably also have potential for applications [17,74,75,78]. Importantly, new attempts to isolate plasmid-dependent phages should be done in order to find phages that are active against commonly circulating conjugative resistance-plasmids as well as to expand our understanding of these special viruses from an evolutionary perspective.

Plasmid-dependent phages can be mass-produced and purified similarly to other phages and hence their administration to animals and humans or introduction into, e.g., agricultural environments or wastewater treatment facilities could interfere with the community-level toolbox of options by which bacteria overcome threats. This, in turn and in the long run, may help alleviate the problems that conjugative resistance plasmids are causing in healthcare. It remains to be determined exactly how these phages should be used in applications. One could envision them being continuously introduced into agricultural settings, animal farming or wastewater processing in an attempt to cause pressure for bacteria to drop their (resistance) plasmids. While it is impossible to target all plasmids simultaneously, the presence of replicating phages that can respond evolutionarily to plasmid evolution could yield positive outcomes. Plasmids in general are minor parasites (and in many cases mutualistic symbionts) of bacteria, but artificially induced over-selection against them via phages can restrain the degrees of freedom in which plasmids naturally operate. It would be a battle in terms of odds: constant use of antibiotics has provided plasmid-borne resistance genes and their plasmid-backbones a highway within the bacterial realm; plasmid-targeting phages could serve as a barricade—not blocking them all, yet reducing the traffic. Naturally, plasmid-dependent phages could be used directly to treat resistant infections in humans. Especially in cases where certain types of plasmids are over-represented in the healthcare setting, these phages can provide a way to kill plasmid-carrying bacteria regardless of their specific host species or strain. This would make it difficult for the plasmids to hide in unconventional (non-pathogenic) hosts, just waiting for the chance to migrate into a rapidly proliferating pathogen once antibiotics are thrown into the equation.

In the end, phages that have evolved to utilize plasmid-encoded features on bacteria are a natural enemy of features that make bacterial systems capable of overcoming threats. Their origin and evolution can help us decipher viral evolution in general and they may allow us to target problematic evolutionary mechanisms in particular.

## Figures and Tables

**Table 1:** Examples of plasmid-dependent bacteriophages.

Family *Genus*	Bacteriophage	Morphology	Size	Genome	Genome Length (bp)	Example Host ^a^	Incompatibility	Phage Attachment Site	References
***Leviviridae***		**icosahedral capsid**	**20–28 nm**	**ssRNA**	**3400–4200**				[51]
*Levivirus*									
	**MS2**					*Escherichia coli*	IncF	Sides of pili	[52]
*Levivirus*									
	**Qβ**					*Escherichia coli*	IncF	Sides of pili	[53]
*Unclassified*									
	**C-1**					*Salmonella typhimurium*	IncC	Sides of pili	[54,55,56]
	**D**					*Escherichia coli*	IncD	Sides of the end of pilus	[57]
	**Hgal1**					*Escherichia coli*	IncH	Sides of pili	[47,54]
	**I** **ɑ**					*Escherichia coli*	IncI_1_	Sides of pili	[58]
	**M**					*Escherichia coli*	IncM	Sides of pili	[50,59]
	**pilH** **ɑ**					*Escherichia coli*	IncHI, IncHII	Sides of pili	[60,61]
	**PRR1**					*Pseudomonas*	IncP	Sides and base of pili	[62,63,64]
	**SR**					*Escherichia coli*	IncS, IncFV	Sides of pili	[65]
	**t**					*Escherichia coli*	IncT	Sides of pili	[66]
***Tectiviridae***		**icosahedral capsid, membrane-containing**	**63 nm**	**dsDNA**					
	**PRD1**				15000	*Escherichia coli*	IncPɑ (IncN, IncW)	Mpf (mating pair formation) complex	[22,67]
***Inoviridae***		**filamentous**	**6–8 nm × 700–2000 nm**	**ssDNA**	**6000–12000**				
	**C-2**					*Salmonella typhimurium*	IncC	Sides of pili	[48]
	**M13**					*Escherichia coli*	IncF	Tip of pili	[29,30]
	**If1**					*Escherichia coli*	IncI	Tip of pili	[68]
	**Ike**					*Escherichia coli*	IncN, IncI_2_	Tip of pili	[69,70]
	**X**					*Escherichia coli*	IncX (IncI_2_, IncM, IncN, IncP-1, IncW)	Tip of pili	[71]
	**Pf3**					*Pseudomonas aeruginosa*	IncP-1	Sides of pili	[63,72]
	**SF**					*Escherichia coli*	IncS, IncFI-V, IncD	Tip of pili	[65]
	**tf-1**					*Escherichia coli*	IncT	Point of pili	[73]
***Unclassified***									
	**J**		40 nm, 18 nm non-contractile tail			*Escherichia coli*	IncJ, IncC, IncD	Sides of pili	[48]

^a^ Alternative hosts may exist as phage host range can be expanded through plasmid host range.

## Data Availability

Not applicable.
